# Transcytosis of T4 Bacteriophage Through Intestinal Cells Enhances Its Immune Activation

**DOI:** 10.3390/v17010134

**Published:** 2025-01-19

**Authors:** Amanda Carroll-Portillo, October Barnes, Cristina N. Coffman, Cody A. Braun, Sudha B. Singh, Henry C. Lin

**Affiliations:** 1 Division of Gastroenterology and Hepatology, University of New Mexico, Albuquerque, NM 87131, USA; 2 Biomedical Research Institute of New Mexico, Albuquerque, NM 87108, USA; october.a.barnes@gmail.com (O.B.); ccoffman1@unm.edu (C.N.C.); cody.braun1@va.gov (C.A.B.); sudha.singh@va.gov (S.B.S.); 3 Medicine Service, New Mexico VA Health Care System, Albuquerque, NM 87108, USA

**Keywords:** bacteriophage, transcytosis, macrophage, TNFα, T4, polarized Caco2

## Abstract

Interactions between bacteriophages with mammalian immune cells are of great interest and most phages possess at least one molecular pattern (nucleic acid, sugar residue, or protein structure) that is recognizable to the immune system through pathogen associated molecular pattern (PAMP) receptors (i.e., TLRs). Given that phages reside in the same body niches as bacteria, they share the propensity to stimulate or quench immune responses depending on the nature of their interactions with host immune cells. While most in vitro research focuses on the outcomes of direct application of phages to immune cells of interest, the potential impact of their transcytosis through the intestinal barrier has yet to be considered. As transcytosis through intestinal cells is a necessary step in healthy systems for access by phage to the underlying immune cell populations, it is imperative to understand how this step may play a role in immune activation. We compared the activation of macrophages (as measured by TNFα secretion) following direct phage application to those stimulated by incubation with phage transcytosed through a polarized Caco2 epithelial barrier model. Our results demonstrate that phages capable of activating TNFα secretion upon direct contact maintain the stimulatory capability following transcytosis. Furthermore, activation of macrophages by a transcytosed phage is enhanced as compared to that occurring with an equivalent multiplicity of directly applied phage.

## 1. Introduction

Bacteriophages (phages) are viruses that target bacteria specifically and require them for propagation (reviewed in [1]). As such, phages can be found in every ecological niche where bacteria thrive, including bacteria-containing microenvironments of mammalian hosts such as the oral cavity and the gastrointestinal system (reviewed in [2]). While phage multiply only in the presence of their bacterial hosts, they still have the propensity to be influenced by, and in turn, influence mammalian cells. Along these lines, the phage interactions in the gastrointestinal system with both the epithelial barrier and, potentially, the underlying immune cells, are ideal for studying the mechanisms by which phage may stimulate immune reactivity.

The effects of phage on the bacteria within the gastrointestinal tract have long been studied both in the context of how they are influenced by their microenvironment to regulate the surrounding bacterial populations [3,4] as well as how they may be applied therapeutically to compete against bacterial pathogens (reviewed in [5,6]). Even though phages do not infect eukaryotic cells, the interest in the potential effects of phages on immune cells arose when capsid proteins on the coliphage T4 were found to contain immunoglobulin-like domains [7,8] capable of binding the β3 integrins of mammalian cells [9] or to mucin glycoproteins [4]. These findings led to the work behind bacteriophage adherence to mucin (BAM)-associated immunity [4,10] and it is now more apparent that there are a variety of ways and places where phage can modulate mammalian immune responses (reviewed in [11,12,13,14]). While evidence has been collected for several types of phages interacting with immune cells or driving specific immune responses in the context of disease [15,16,17,18,19,20], the body of literature on T4 coliphage is the most extensive.

T4 coliphage is a commensal, lytic phage of the gastrointestinal microbiome that infects *Escherichia coli* by initially binding to the bacterial lipopolysaccharide structure [21]. While the infective life cycle of T4 has been worked out [21], the interactions and effects of this phage on host cells are more complicated and less clear. Phages have been found in the bloodstream and extraintestinal sites in healthy and unhealthy individuals indicating their ability to access these sites [22,23,24,25,26,27]. This is supported by in vitro data demonstrating the ability of a multitude of phage types, including T4, to translocate across several types of cellular barriers [28,29]. Furthermore, in vivo data have demonstrated that specific adaptive immune responses may be triggered by T4, including the generation of T4 specific antibodies and the contribution to T-cell specific immunity [30,31,32,33,34]. However, in vitro application of T4 to specific immune cells (PBMCs and dendritic cells) with either an intact phage or its most immunogenic extracellular proteins (Hoc, Soc, and gp23) have shown that neither a pro- nor an anti-inflammatory response is triggered [32,35]. Since luminal phages can only access immune cells in the lamina propria by first crossing the intestinal barrier by transcytosis, whether a phage activates immune cells differently following transcytosis is not known.

To understand how interactions of phage with intestinal cells might drive immune responses, we utilized TNFα secretion from mammalian macrophages (RAW264.7 cells) as our outcome to assess the phage activation of a host’s innate immune response. We established this readout utilizing T4r+ bacteriophages to compare their stimulation of macrophages either directly applied or transcytosed across polarized Caco2 cells.

## 2. Materials and Methods

*Bacteriophage and cell lines*. The coliphage T4r+ (T4) and its host bacteria (*Escherichia coli* B; EcB) were purchased from Carolina Biological Supply, Burlington, NC and ATCC, Manassas, VA, respectively. *Limisilactobacillus plantarum* (*L. plantarum*) phage 8014-B2 and its host bacteria, *L. plantarum*, were purchased from ATCC. Phage stocks were generated as previously described [36]. Briefly, outgrowth on the host strain was followed by purification via filtration and cesium chloride gradient centrifugation. Phage were dialyzed and stored in an SM Buffer (200 mM NaCl_2_, 10 mM MgSO_4_, 50 mM Tris-HCl, pH 7.5) and enumerated using plaque overlay assay [37]. A polarized Caco2 and RAW264.7 cell lines (ATCC) were used for testing the effect of transcytosis across the small intestinal epithelial barrier and for measuring immune activation (TNFα production in response to phage stimulation), respectively. Caco2 cells were maintained in Caco2 medium (Dulbecco’s Modified Eagle Medium (DMEM), 20% Fetal Bovine Serum (FBS), 1% Penicillin-Streptomycin (P/S), 1% non-essential amino acids; ThermoFisher, Waltham, MA, USA) and RAW264.7 cells were grown in RAW medium (DMEM, 10% FBS, 1% P/S).

*Transcytosis across intestinal cells.* Barriers mimicking the small intestine were generated by growing differentiated and polarized Caco2 cells as described by Natoli et al. [38]. Briefly, Caco2 were seeded on 0.4 μm transwells (Corning, Corning, NY, USA) at a density of 2.5 × 10^5^ cells/transwell (500 μL medium in apical well and 1mL in basolateral well) and grown for 18–21 days with media exchange every 3–4 days. The final Caco2 cell number after polarization was determined by trypsinizing cells from the filter surface and performing a cell count with hemacytometer. Average of these counts (n = 3) showed approximately 1 × 10^6^ cells/filter at day 21. Antibiotics were removed from the cells when the media was changed prior to assays. T4 was added to the apical surface of barriers at a multiplicity of 10 or 100. After 24 h incubation with phage, basolateral supernatants were collected and separated for subsequent use in macrophage stimulation (800 μL), or plaque overlay assays (200 μL).

*Macrophage stimulation*. For testing the immune response to phage stimulation, RAW264.7 cells were seeded in 12 well plates at a density of 0.5 × 10^6^ cells/well in RAW medium (for direct stimulation) or antibiotic free Caco2 medium (for stimulation with transcytosed phage) for overnight incubation. The media was removed and replaced with a fresh RAW medium or 800 μL of basolateral medium, and cells were incubated for 24 h at 37 °C, 5% CO_2_. Supernatants of the RAW medium were collected and the concentration of secreted TNFα was measured using ELISA MAX Deluxe Set Mouse TNFα kits (Biolegend, San Diego, CA, USA). A total of one hundred μL of macrophage supernatants following stimulation were assayed in triplicate alongside a standard curve to quantify the concentration of TNFα present. Colorimetric readings were taken on a plate reader at 450 nm. Values are presented as mean ± standard error of the mean (SEM).

*Intestinal barrier function*. Before and after the addition of a phage, transepithelial electrical resistance (TEER) was measured for all wells using the EVOM Epithelial Volt/Ohm Meter 3 (World Precision Instruments, Sarasota, FL, USA). After basolateral supernatants were collected, the media was replaced and transwells were exposed to 0.2 mg/mL Fluorescein isothiocyanate-dextran (4kDa Fluorescein isothiocyanate (FITC)-dextran, MilliporeSigma, Burlington, MA, USA) solution (in Caco2 medium) for 1 h at 37 °C, 5% CO_2_. After incubation, 100 μL of basolateral medium was assayed in duplicate within a fluorescence microwell plate (Corning) on a plate reader with reads taken at 450 nm.

*Immunofluorescence*. A T4 phage was labeled with either SYBR Gold (SG, nucleic acid, ThermoFisher) or both SG and Alexa647-NHS ester (A647, capsid, ThermoFisher). Briefly, T4 stocks were quantitated using plaque overlay assays and incubated with a 1x concentration of SYBR Gold in the dark for 30 min with occasional gentle agitation. For reactions where both nucleic acid and capsids were labeled, phage solutions were exchanged to 40 mM HEPES buffer, pH 8 using 10,000 (10K) molecular weight cutoff (MWCO) centrifugal filters (MilliporeSigma) and incubated with 1x SG and an A647 concentration to allow for a degree of labeling of 1–5 dye molecules/phage particle. Labeling reactions were incubated in the dark for 30 min with occasional, gentle agitation. Free dye removal and washes were performed with 10K MWCO centrifugal filters with final, cleaned concentrate brought up to original volume in an SM Buffer. After 24 h incubation with labeled phage, polarized Caco2 filters were fixed with 4% paraformaldehyde (PFA; Electron Microscopy Sciences, Hatfield, PA, USA), washed with PBS, and mounted with DAPI-containing ProLong Gold (ThermoFisher). Samples were imaged on a Fluoview Olympus confocal microscope (Olympus Life Science, Center Valley, PA, USA) using a 63x objective.

*Image processing and Data analysis*. Confocal microscopy images were processed and analyzed using FIJI freeware (ImageJ, [39]). To determine the number of cells within a field of view containing phage, Z-stacks were z-projected with max intensity and channels were overlaid (cyan- nuclear stain (DAPI), green- SG, phage nucleic acid, red- phage capsid). The total number of nuclei and the number of nuclei with close proximity to phage material were counted, and the percent which were phage positive was determined.

*Data analysis*. Data processing, graphing, and statistical analyses were conducted using GraphPad Prism software v.10.4.1 (GraphPad, San Diego, CA, USA). Descriptive statistics (mean and standard error of mean (SEM)) and Brown–Forsythe and Welch one-way ANOVA tests with Dunnett’s multiple comparisons test were performed to identify statistical significance.

## 3. Results

### 3.1. Directly Applied T4 Stimulated TNFα Production Macrophages in a Concentration-Dependent Fashion

**Figure 1:**
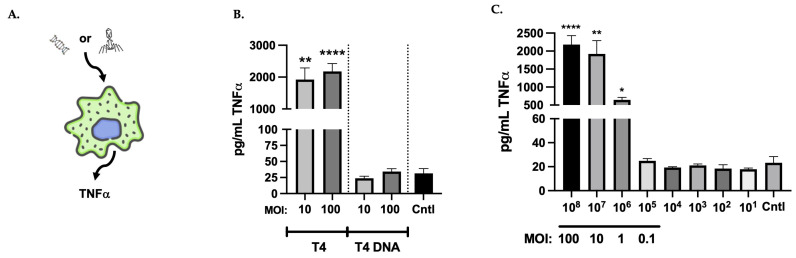
T4 bacteriophage activates TNFα secretion in murine macrophages. (**A**) Experimental diagram of application of either intact T4 or T4 DNA to RAW264.7 cells with TNFα secretion as evidence of activation. (**B**) Quantitation of TNFα secreted by RAW264.7 in response to multiplicity of 10 or 100 T4 (left two columns) and multiplicity of 10 or 100 equivalents of T4 DNA (middle two columns) as compared to RAW264.7 alone (Cntl). (**C**) Quantitation of TNFα from RAW264.7 incubated with a titration of intact T4 as compared to RAW264.7 alone (Cntl). Multiplicity of 100 was highest concentration used (10^8^). Columns represent mean values with standard error of the mean (SEM). **** = <0.00001, ** = 0.001–0.009, * = 0.01–0.09.

To determine if a directly applied T4 phage could stimulate TNFα secretion from RAW264.7 macrophages (RAW cells), phages were added to RAW cells at a multiplicity of 10 or 100 and incubated for 24hr to give maximal time for response (Figure 1A). The concentration of TNFα in supernatants in response to phage exposure was quantitated with ELISA. A standard curve was generated at the same time. Direct application of intact T4 resulted in high levels of TNFα secretion at both multiplicities tested with multiplicity of 10 inducing 1922 ± 366 pg/mL (*p* = 0.001) and multiplicity of 100 inducing 2180 ± 248 pg/mL (*p* < 0.0001) TNFα as compared to media only controls (31.6 ± 7.3 pg/mL) (Figure 1B, T4 columns). As macrophages possess several receptors capable of recognizing pathogen associated molecular patterns (PAMPs) such as viral nucleic acid, we also examined if purified T4 DNA was able to stimulate TNFα secretion in RAW cells in a manner similar to an intact phage. With the known concentration of DNA within a single T4 capsid, ~57 ng, multiplicity of 10 and 100 DNA equivalents were calculated and added to RAW cells for 24 h incubation. Analysis of RAW supernatants after direct DNA application demonstrated that TNFα secretion was not significantly different than media only controls with 23.9 ± 3.2 pg/mL for multiplicity of 10 equivalent and 34.3 ± 4.4 pg/mL for multiplicity of 100 equivalent (Figure 1B, T4 DNA columns).

These results, in combination with the literature suggesting the importance of phage titer in stimulation of mammalian immune responses [12,14,20,40], prompted an examination to determine the cutoff concentration of T4 for activation. Volumes of serially diluted T4 starting at a high concentration of multiplicity 100 (10^8^) were directly applied to RAW cells for 24 h incubation. Supernatants were again analyzed with ELISA and demonstrated a titration of activation with increased dilution (Figure 1C). The stimulation of TNFα secretion by intact T4 was significantly above control values until dilution of phage reached 10^5^ phages/mL, which corresponds to a multiplicity of 0.1 (10^8^ = 2180 ± 247.9 pg/mL, *p* < 0.0001; 10^7^ = 1922 ± 366.4, *p* = 0.004; 10^6^ = 643.7 ± 70.8 pg/mL, *p* = 0.03; and 10^5^ = 24.9 ± 1.8 pg/mL, *p* = ns (not significant) vs. control (Cntl) = 23.26 ± 5.2).

### 3.2. T4 Transcytosis Occurred at Low Level with No Effect on Intestinal Barrier

**Figure 2:**
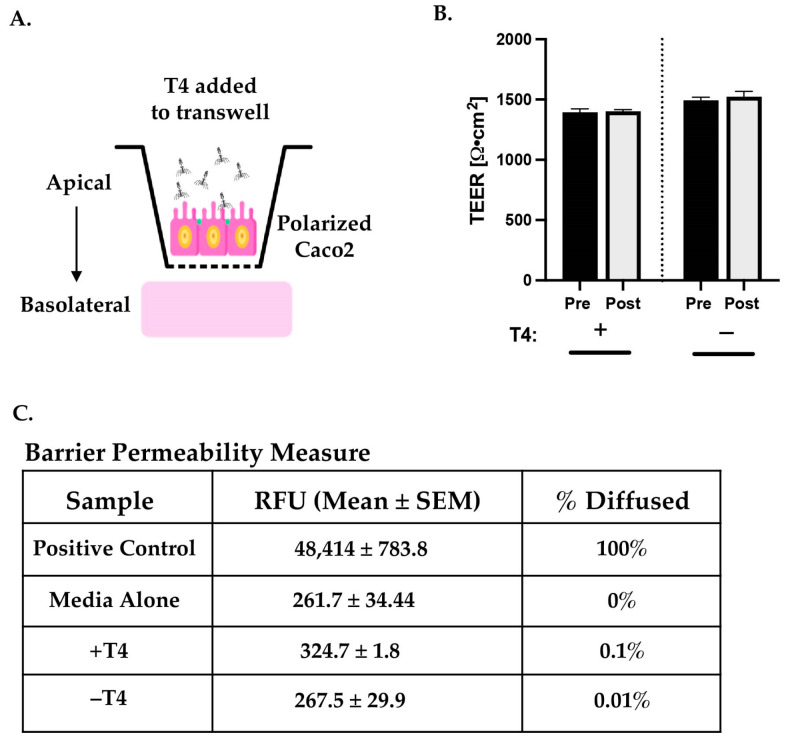
Application of T4 does not affect barrier function. (**A**) Diagram of in vitro application of T4 to transwells containing small intestinal barrier models (polarized Caco2). (**B**) Comparison of TEER measurements pre- and post-experiment from transwells with T4 applied (+ columns) or barriers alone (− columns). Columns represent mean values with standard error of the mean (SEM). (**C**) Results from FITC flux assay with mean relative fluorescence measures ± SEM from the basolateral media of barriers with (+T4) or without (−T4) phage application and the calculated percent of flux occurring for each group of samples.

T4 has been shown to transcytose through several models of epithelial barriers in vitro [28], and the expectation was that this phage would also transcytose across a monolayer of polarized Caco2 cells, which mimics the small intestinal epithelium. T4 at a multiplicity of 10 or 100 was applied to the apical surface of polarized Caco2 cells (days 18–21) grown on transwell filters (Figure 2A). To ensure that T4 application was not detrimental to the Caco2 barriers, measurements of both TEER and FITC-dextran flux were performed. The resistance of barriers, as measured by TEER, is correlated to the impermeability of cellular tight junctions. In our experiments, TEER remained high after incubation with T4, on par with control barriers that were not exposed to T4 (Figure 2B). Barriers exposed to T4 had a mean measure of 1393 ± 29.7 Ω•cm^2^ prior to incubation and 1401.4 ± 14.1 Ω•cm^2^ after incubation as compared to control barriers (no T4) which started with 1493 ± 26.1 Ω•cm^2^ and ending at 1523 ± 44.7 Ω•cm^2^.

To determine whether T4 caused increased permeability in treated intestinal barriers, allowing for paracellular flux (movement through junctional spaces), we followed up with the use of a FITC flux assay. After 24 h T4 incubation, diffusion of 4 kDa FITC-dextran from the apical portion of the transwell through the barrier to the basolateral portion was monitored by detection of relative fluorescence (RFU) within the basolateral medium where increased RFU would indicate a “leaky” barrier. Results of FITC flux assays showed that barriers retained their tight junctions despite incubation with T4 (Figure 2C). To determine the percent of fluorescence that diffused through the barrier, the positive control value (highest amount of FITC that could diffuse through; mean RFU = 48414) was set to 100%. The media alone value (mean RFU = 261.7) was subtracted from the sample means with (+T4) and without (−T4) phage added, and then the percent RFU relative to the positive control was determined. Calculations showed that untreated, polarized barriers allowed 0.01% of the total FITC through the barrier, and treated, polarized barriers allowed a passage of 0.1%. These data, taken in conjunction with the TEER data, showed that incubation with a multiplicity of 100 of T4 phage for a 24 h period was not detrimental to the barrier integrity.

### 3.3. T4 Transcytosed Across Polarized Caco2 Cells at a Low Level

**Figure 3:**
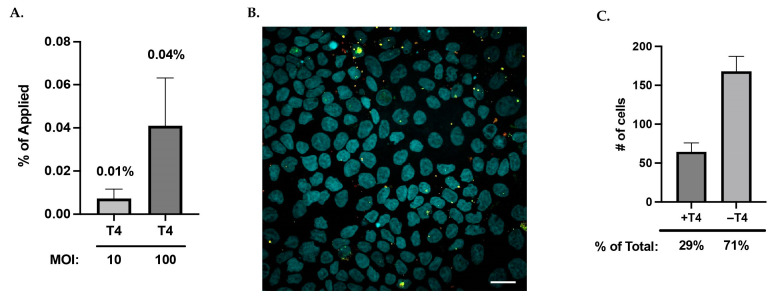
Low percentage of applied T4 transcytose through a limited number of barrier cells. (**A**) Calculated percentage of applied, active T4 that is transcytosed through polarized Caco2 after 24 h as measured by plaque overlay assay. (**B**) Representative overlayed Z-projection microscopy image (60×) of Caco2 barrier cells containing labeled T4 (nuclei = DAPI, pseudocolored cyan; T4 nucleic acid = SG, pseudocolored green; T4 capsid = AF647, pseudocolored red). Yellow pixels indicate colocalization of T4 labels. Image has been brightness/contrast enhanced, and scale bar is 20 μm. (**C**) Comparison of the mean number (± SEM) of Caco2 cells within a field of view found with labeled phage (+T4) to the number of Caco2 cells with no phage (−T4). Calculations include 12 fields of view across five experiments.

Previously, active T4 transcytosis through an in vitro cell model of the colonic epithelium (Caco2, 3–4-day confluency) found approximately 0.1% of the apically applied phage transcytosed to the basolateral side [28]. To determine the % of active T4 that transcytosed across polarized Caco2 cells (small intestinal model, 21-day confluency), we enumerated active phages applied to the apical surface and the active phages within the basolateral media by performing plaque overlay assays (Figure 3A). We found that active T4 transcytosed at an even lower level than that seen in the colonic model; specifically, T4 at a multiplicity of 100 (10^8^) resulted in transcytosis of 0.04 ± 0.02% of the applied phage and T4 at a multiplicity of 10 (10^7^) resulted in transcytosis of 0.01 ± 0.004%. Confocal microscopy was performed to visualize labeled T4 within the polarized Caco2 cells (Figure 3B) demonstrating that the uptake of T4 was not uniform, with some cells appearing to have a higher affinity for uptake than others. Calculation of the number of cells containing labeled T4 DNA after 24 h incubation found that only 29 ± 5.3% of cells within a field of view contained phage (Figure 3C). Furthermore, cells positive for transcytosis tended to be clustered together suggesting these cells may have a physiological difference that increased the likelihood for transcytosis.

### 3.4. Transcytosed T4 Stimulate More TNFα Secretion than Directly Applied T4

**Figure 4:**
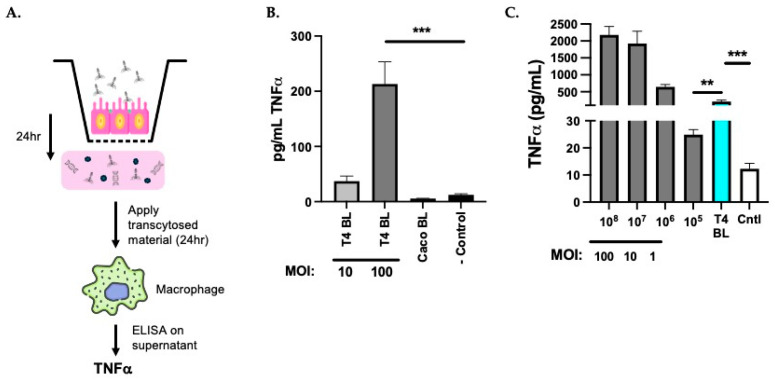
Transcytosed T4 induces TNFα secretion from murine macrophages better than an equivalent concentration of directly applied T4. (**A**) Experimental diagram of application of T4 for transcytosis followed by application of basolateral medium to macrophages for TNFα induction. (**B**) Comparison graph of mean (± SEM) concentration of TNFα quantitated from RAW 264.7 cells treated with the basolateral (BL) medium from barriers with T4 applied as compared to RAW 264.7 cells alone (-Control) or within the BL medium itself of T4-treated Caco2 barriers (Caco BL). (**C**) Comparison of the mean (± SEM) secreted TNFα (pg/mL) from RAW 264.7 cells treated with directly applied T4 (10^8^ through 10^4^) to that of RAW cells treated with the BL fraction from barriers with a multiplicity of 100 T4 applied (blue bar; transcytosed concentration equivalent to 10^4^). The control medium was from untreated RAW 264.7 cells (Cntl). *** = 0.0001–0.0009 and ** = 0.001–0.009.

While stimulation of TNFα secretion from RAW cells by T4 occurred after direct application, it does not match the steps that must take place in vivo; specifically, gastrointestinal phages need to traverse the epithelial barrier (in healthy individuals) prior to interacting with immune cells in the lamina propria. As such, we questioned whether transcytosis across intestinal cells might influence the effectiveness of phage stimulation of macrophages. To investigate this, we applied T4 at either a multiplicity of 10 or 100 to the apical surface of a polarized monolayer of Caco2 cells, allowed 24 h for transcytosis to occur, and then applied the basolateral medium to RAW cells for an additional 24 h before screening the supernatant for TNFα secretion (Figure 4A). A time of twenty-four hours was selected to allow for transcytosis of a measurable amount of T4 and for a response by RAW cells.

Analysis of the RAW supernatant (Figure 4B) revealed that transcytosed material from the application of T4 at a multiplicity of 10 stimulated low levels of TNFα secretion (37 ± 9.5 pg/mL), as compared to what is secreted by RAW cells upon exposure to basolateral medium from untreated Caco2 barriers (-Control; 12.32 ± 2 pg/mL). This difference was not found to be statistically significant (*p* = 0.3). In comparison, exposure to basolateral medium from barriers with a multiplicity of 100 T4 applied stimulated significantly higher TNFα secretion from the RAW cells (213.2 ± 40.2 pg/mL; *p* = 0.003). This measurable amount of TNFα is strictly from the RAW cells as the basolateral media from treated Caco2 cells (Caco BL) did not contain TNFα in response to incubation with T4 at a multiplicity of 100 (5.579 ± 0.5 pg/mL). The calculation of active T4 found to transcytose polarized Caco2 barriers at a multiplicity of 100 (0.04% of applied phage) is equivalent to 10^5^ T4 found within the basolateral media, and this is the concentration of phage to which the RAW cells are subsequently exposed. As such, we compared the TNFα secretion by the RAW cells upon direct exposure to 10^5^ T4 vs. exposure to 10^5^ transcytosed T4 and found that the amount of TNFα secreted by the RAW cells incubated with basolateral media is significantly higher than that produced from direct application of the comparable amount of T4 (213.2 ± 40.2 pg/mL vs. 24.9 ± 1.8 pg/mL; *p* = 0.002) (Figure 4C). These data demonstrate that transcytosis of T4 through an in vitro model of the small intestinal epithelium enhanced T4 activation of the immune response by RAW cells, as measured by TNFα secretion.

### 3.5. Transcytosis Does Not Change Macrophage Response to Non-Immunostimulatory Phage

**Figure 5:**
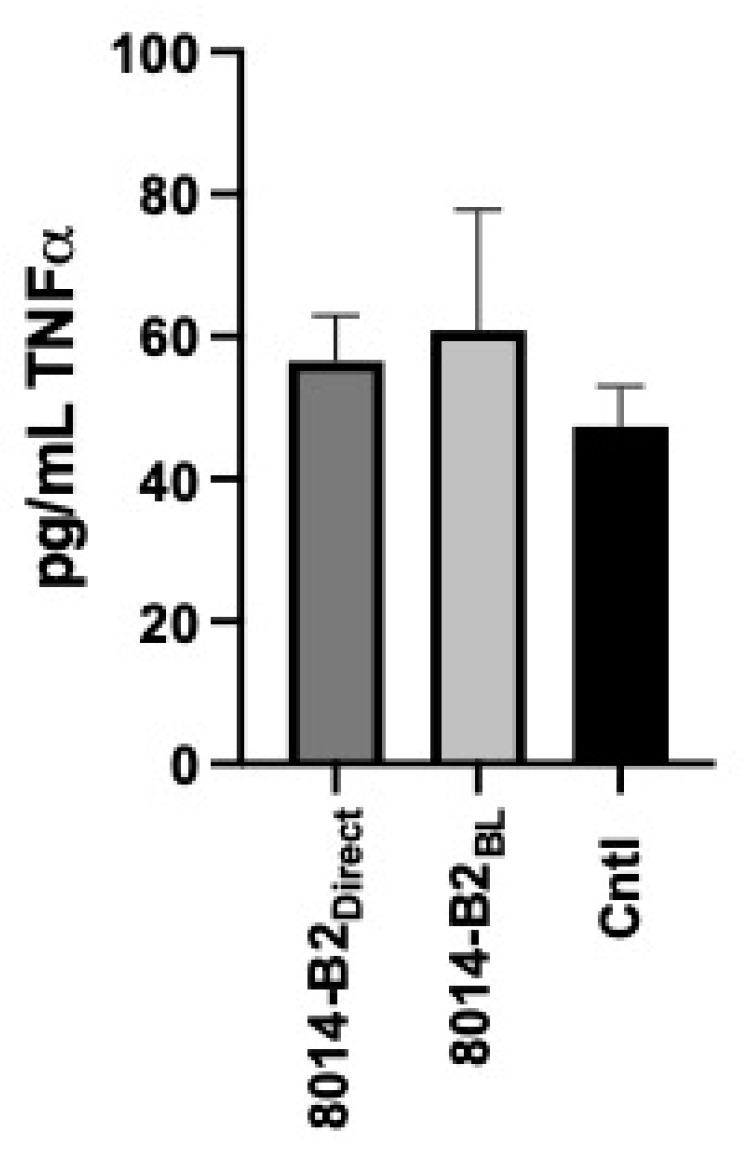
Transcytosis alone is insufficient to make phage immunostimulatory. Comparison of secreted TNFα concentrations (pg/mL; mean ± SEM) from untreated RAW264.7 cells (Cntl) to that found from cells treated to direct application of the *L. plantarum* phage 8014-B2 (Direct) or transcytosed 8014-B2 (BL).

While transcytosis through polarized Caco2 cells enhanced activation of the RAW macrophages by T4, we questioned whether transcytosis alone was sufficient to functionally change any transcytosed phage to be more immunostimulatory. We found during testing of several different phages that, unlike T4, the *Limosilactobacillus plantarum* bacteriophage, 8014-B2, does not induce TNFα secretion by RAW macrophages when directly applied (Figure 5; 8014-B2_Direct_ vs. Cntl= 56.5 ± 6.4 pg/mL vs. 47.4 ± 5.6 pg/mL). To see whether transcytosis might modify this non-immunostimulatory phage to make it immunostimulatory, a multiplicity of 100 of 8014-B2 was applied to polarized Caco-2 barriers and allowed to incubate for 24 h. Similar to the T4 experiments, the basolateral medium was collected and applied to RAW macrophages for an additional 24 h incubation period. RAW supernatants were then tested for TNFα with ELISA. Transcytosis of 8014-B2 did not result in an increased secretion of TNFα by the treated RAW cells (Figure 5; 8014-B2_BL_; 60.9 ± 17 pg/mL) suggesting that transcytosis is not sufficient to change a phage from non-stimulatory to stimulatory.

## 4. Discussion

Despite early belief that bacteriophages were innocuous from a clinical standpoint, as they specifically infect bacteria rather than mammalian cells [41], it has instead turned out that the dynamics of interactions between bacteriophages and mammalian host cells are much more complex. In vitro assays have demonstrated both pro- and anti-inflammatory responses (reviewed in [12,14,42]), while in vivo administration has shown the development of anti-phage antibodies [32,43], the induction of T-cell responses [44,45], and the modulation of the immune response to both the benefit and detriment of the phage’s bacterial host [46,47,48,49]. Indeed, it has even been demonstrated that response to any given phage may be further modulated by the specific species of bacterial host and the phage titer involved [50,51,52]. But, prior to an interaction with the immune system, a critical step exists in that any gastrointestinal bacteriophage needs to move past the epithelial barrier (small intestinal or colonic) in order to access the niches where immune cells reside.

The structure of the gastrointestinal barrier differs between the large intestine and the small intestine, descriptions of which can be found in several thorough reviews [53,54,55]. Herein, we focus our research on dynamics of a representative intestinal phage (T4) with an in vitro model of the small intestinal barrier consisting of polarized Caco2 cells grown on transwell filters for 18–21 days. Within the distal small intestine, phages, which have no means for self-propulsion, exist in a context of high bacterial density (on the order of 10^9^ bacteria/mL) [56] and thinner mucin coverage [57] allowing for the potential of close proximity with the epithelial barrier, more so than would be possible within the colonic microenvironment [58,59]. However, the tight junctions of the epithelial barrier in the distal ileam are such that only particles that are <13 nm are capable of moving between cells (paracellularly) [60], meaning that transcytosis (or movement through the epithelial barrier) is the more likely mechanism for phages, which are larger than 13 nm, to access the underlying tissues. Indeed, several reports have described transcytosis of a variety of phages through in vitro epithelial and endothelial barriers [28,29,61], suggesting that this is a likely route for phage access to extraintestinal niches in vivo. These reports have described a number of factors concerning bacteriophage transcytosis including: Phages of different shapes and sizes are transcytosed preferentially in an apical to basolateral direction [28]. Uptake of phage occurs early after exposure with surface adherence within 30 s and internalization visualized within 10 min [29]. Endosomal trafficking involves Golgi-associated vesicles and some breakdown of phage [28]. A majority of internalized phage remain intact and functional [29]. And, a phage that is transcytosed to the basolateral represents a fraction of what was applied in a dose dependent manner, meaning that if more phage is present apically, more will be found transcytosed over time [28,29].

Our data are supported by these findings, although it is possible that phage transport through the polarized Caco2 cells occurs through different pathways than those previously described. What is particularly intriguing is that our results demonstrate that transcytosed T4 displays enhanced immunostimulatory properties (initiating TNFα secretion in macrophages) as compared to an equivalent concentration of non-transcytosed T4. However, transcytosis alone was insufficient to make a non-stimulatory phage (i.e., 8014-B2) more stimulatory, suggesting either that there are certain structural differences between phages that result in modification during transcytosis or that modification occurs regardless but is only stimulating in the context of other phage-specific motifs.

In a clinical context, we posit that the modification of certain phages through transcytosis for enhanced immune reaction could serve to benefit the host. It has been reported that the T4 phage does not induce a proinflammatory response [32,35], but does induce antibody production [30,31,32]. In the healthy gastrointestinal system, the bacteria to phage ratio is reported to be low (1:1) [62] and the number of phages that are immunostimulatory are also likely to be limited given the literature that supports anti-inflammatory phage-driven responses [14]. Thus, the concentration of transcytosed-immunostimulatory phage would be even lower meaning that interaction events with tissue resident macrophages would be likely limited and potentially drive the more beneficial effects associated with TNFα secretion (e.g., increased barrier integrity) [63,64]. If the intestinal system becomes disrupted, such as occurs with dysbiosis (e.g., Small Intestinal Bacterial Overgrowth, Irritable Bowel Syndrome, or bacterial infection), the disease state can be associated with significant increases in any given phage [65,66,67], including those that are proinflammatory. In these instances, the higher titers of proinflammatory phages in the lumen would be reflected in the transcytosed levels, and the induction of TNFα secretion by intestinal macrophages would be more frequent as the likelihood of interaction between transcytosed immunostimulatory phages and macrophages is increased. Given the potency of TNFα to further drive inflammation, it is possible that the induction of peritoneal macrophages in the context of the expansion of a proinflammatory phage population would work to initiate and enhance inflammation associated with dysbiosis/disease. Better understanding of the mechanisms of increased immunostimulation by transcytosed phages and the potential contributions to intestinal homeostasis or disease progression require both in vitro and in vivo follow-on studies with clear consideration as to the complexity and nature of the potential interactions.

## Data Availability

The raw data supporting the conclusions of this article will be made available by the authors on request.
